# Green first derivative synchronous spectrofluorimetric determination of lacidipine and its acid degradation product in plasma and mixtures

**DOI:** 10.1038/s41598-025-11341-y

**Published:** 2025-07-17

**Authors:** Khalid A.M. Attia, Mohamed A. Hasan, Abdalla M. El-Shanawany

**Affiliations:** 1 Pharmaceutical Analytical Chemistry Department, Faculty of Pharmacy, Al-Azhar University, Nasr City, 11751 Cairo Egypt; 2 Pharmacist at Al-Azhar University, Cairo, Egypt

**Keywords:** Lacidipine, Spiked human plasma, Synchronous fluorescence, Pharmaceutical Preparation, Biochemistry, Chemistry

## Abstract

One of the main objectives of this study is to find an analysis method that has a low environmental and health impact and contributes to achieving sustainability. A simple and sensitive method was established for determination of lacidipine in pure active pharmaceutical ingredient, pharmaceutical formulation and spiked human plasma in presence of its acid induced degradation product. The native emission was measured at 430 nm after the excitation at 281 nm. A 0.5% Tween-80 solution was used to enhance fluorescence intensity. Peak amplitude of the first derivative synchronous was measured at 409 nm by using constant wavelength difference (Δλ = λ_em_ - λ_ex_ = costant) which equal 160 nm. The regression plot of the proposed first derivative synchronous spectrofluorimetric method was found to be linear over the range of 50–300 ng/mL with a determination coefficient equals 0.9997, limit of detection (LOD) was 14.51 ng/mL but limit of quantification (LOQ) was 43.97 ng/mL.

**Supplementary Information:**

The online version contains supplementary material available at 10.1038/s41598-025-11341-y.

## Introduction

**Fig. 1:**
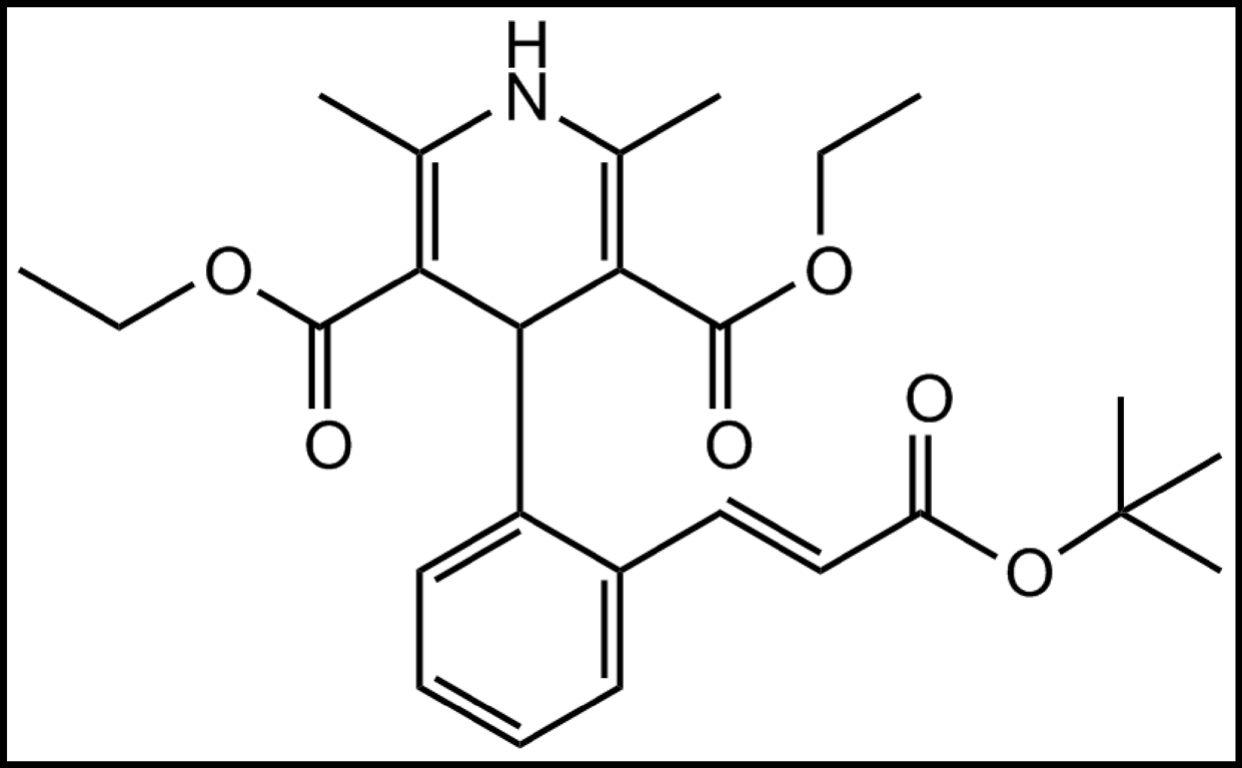
The chemical structure of lacidipine.

Lacidipine is diethyl (E)−4-{2-[(tert-butoxycarbonyl)vinyl]phenyl}−1,4-dihydro-2,6-dimethylpyridine-3,5-dicarboxylate as in Fig. 1. Lacidipine is a calcium channel blocker as antihypertensive drug. The reported analytical methods for determination of lacidipine in biological samples and pharmaceutical formulations are LC-MS/MS^1–3^, HPLC^4–13^, UV spectrophotometry^14–19^ and other methods for determination^20–25^. The British Pharmacopeia showed that it melts at 178^o^c, it describes gas chromatographic method for separation and its chromatographic conditions.

Fluorescence spectrometry is an essential analytical technique for chemical quantification because of its great sensitivity. Overlapping broadband spectra may cause problems with multicomponent analysis’s selectivity^26,27^. The frequent occurrence of fluorescence spectrum overlapping problems limits the use of spectrofluorimetry for multicomponent measurement. The synchronous detection mode, which looks at the excitation and emission spectra at the same time, might help with this issue. This method enhances band resolution by decreasing overlap and spectral band narrowing. Furthermore, the resolution and selectivity of synchronous spectra can be improved by applying mathematical derivatization techniques such as first- or second-order derivatives. When combined, these techniques increase the clarity and precision of the analysis^28,29^.

The fluorescence spectra of lacidipine and its degradation product showed a considerable overlap when they were first observed. The authors are therefore urged to create a first derivative synchronous spectrofluorimetric method that will allow the drug and its degradation product to be determined simultaneously in different matrices.

## Experimental

### Instrumentation

Software and instruments that had been used in this proposed study include; Spectrofluorometer Jasco FP-6200 (Japan). UV lamp (Germany). Rotary evaporator Scilogex-RE 100-pro (USA). Hot plate Torrey pines Scientific (USA), pH meter Jenway 3510 (England).

### Materials and reagents

#### Pure samples

Lacidipine (99.65%) was kindly supplied by Egyphar for pharmaceutical products, Egypt.

#### Pharmaceutical formulation

Lacirex 4 mg tablets (batch no. 4019 A) which were used in this proposed study, purchased from pharmacy.

#### Chemicals

All reagents which used during this proposed study were HPLC grade to avoid any effect of impurities, reagents from (Sigma-Aldrich, Germany) are acetone, acetonitrile, ethanol, methanol, 1-propanol, tetrahydrofuran, boric and acetic acids, from (Merck, Germany) are sodium dodecyl sulphate (SDS) and tween-80, from (Winlab, UK) is cetyltrimethylammonium bromide (CTAB), from (Riedel–deHäen, Germany) is phosphoric acid, from (El–Nasr, Egypt) methyl cellulose (MC) and from (FlukaChemie, Germany) β-cyclodextrin (β‐CD).

### Standard solutions

The concentration of lacidipine stock standard solution was (1 mg/mL) but the concentration of working solution was (1 µg/mL) by dilution of proposed stock solution with methanol.

### Standard solution of lacidipine acid induced degradation product

**Fig. 2:**
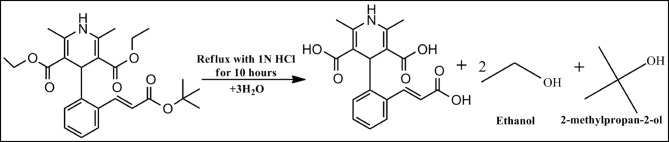
Proposed degradation pathway of lacidipine.

A total of 100 mg of pure lacidipine was dissolved in a minimal volume of methanol in a 100 mL volumetric flask. Then, 50 mL of 1 N hydrochloric acid was added. The mixture was heated under reflux for 10 h to induce acid hydrolysis. After cooling, the solution was neutralized using 1 N sodium hydroxide until pH 7 was reached. The resulting mixture was evaporated under vacuum to dryness. The residue was extracted three times using 25 mL of methanol each time. The combined extracts were filtered to remove any insoluble materials. The filtrate was transferred to a 100 mL volumetric flask and diluted to volume with methanol to obtain a stock degradation solution equivalent to 1 mg/mL of lacidipine. A working solution (1 µg/mL) was prepared by further dilution of the stock solution with methanol. The proposed degradation pathway is illustrated in Fig. 2.

### Procedures

#### Construction of a calibration curve

Aliquots equivalent to 0.5–3 µg of lacidipine were transferred into separate 10 mL volumetric flasks. After addition of 1.5 mL of Britton–Robinson buffer (pH 5.0) and 1 mL of 0.5% tween-80, the volume was completed to 10 mL with distilled water. The resulting concentrations ranged from 50 to 300 ng/mL. Synchronous fluorescence spectra were measured at a constant wavelength difference (Δλ = 160 nm), and the first derivative amplitude was recorded at 409 nm.

#### Analysis of laboratory prepared mixtures

The procedure of the proposed method was applied using aliquots of lacidipine solution (1 µg/mL) containing (2.5–0.5 µg) with aliquots of its acid induced degradation product solution (1 µg/mL) containing (0.5–2.5 µg), hence regression equation calculated the concentrations of lacidipine.

#### Analysis of pharmaceutical Preparation

Ten Lacirex 4 mg tablets were finely powdered. A portion of the powder equivalent to 10 mg of lacidipine was transferred into a 100 mL conical flask with a screw cap. Then, 75 mL of methanol was added. The mixture was vigorously shaken for 15 min, followed by sonication for 30 min. After that, the volume was completed to 100 mL with methanol to obtain a stock solution containing 100 µg/mL of lacidipine. This solution was further diluted with the same solvent to prepare a working solution of 1 µg/mL.

**Procedure for Spiked Human Plasma** Human plasma was obtained from the tumor unit at Al-Azhar University (Cairo, Egypt). In 10 mL centrifuge tubes, 1 mL of drug-free human plasma was transferred. Aliquots from lacidipine and its degradation product standard solutions (1 µg/mL) were then added. Protein precipitation was performed by adding 5 mL of methanol. The samples were vortexed and centrifuged at 400 rpm for 30 min. The resulting protein-free supernatants were evaporated to dryness using a rotary evaporator under vacuum. The residues were reconstituted with 1.5 mL of Britton–Robinson buffer (pH 5) and 1 mL of 0.5% tween-80 solution. The volume was then completed to 10 mL with distilled water and mixed well. The final solutions were filtered through 0.45 μm membrane filters to remove suspended materials and protein residues. To evaluate the method’s ability to detect lacidipine in spiked plasma, quality control (QC) samples were prepared to reduce potential matrix interferences. These samples were categorized as low (LQC), medium (MQC), and high (HQC) concentrations:

- **LQC** was set at three times the lower limit of quantification (LLOQ).
- **MQC** was prepared at 30–50% of the highest concentration in the calibration range.
- **HQC** represented 70% of the highest concentration.

All QC samples were prepared and analyzed using the general procedure, and the percentage recovery (%R) was calculated accordingly. A separate calibration curve was constructed for plasma analysis to eliminate potential matrix effects caused by endogenous plasma components. Standard lacidipine solutions were spiked into blank human plasma and subjected to the full sample preparation procedure (protein precipitation, centrifugation, evaporation, and reconstitution). The regression equation derived from these spiked plasma samples was exclusively used to calculate lacidipine concentrations in unknown plasma samples as in Table 5.

#### The reported method

The published method^30^ based on UV spectrophotometric method of lacidipine in tablets and bulk, it depend on the reaction of lacidipine with HCl, FeCl_3_ and K_3_Fe(CN)_6_ to produce bluish green colored chromogen with an absorption maximum at 740 nm.

## Results & discussion

### Spectral characteristic

**Fig. 3:**
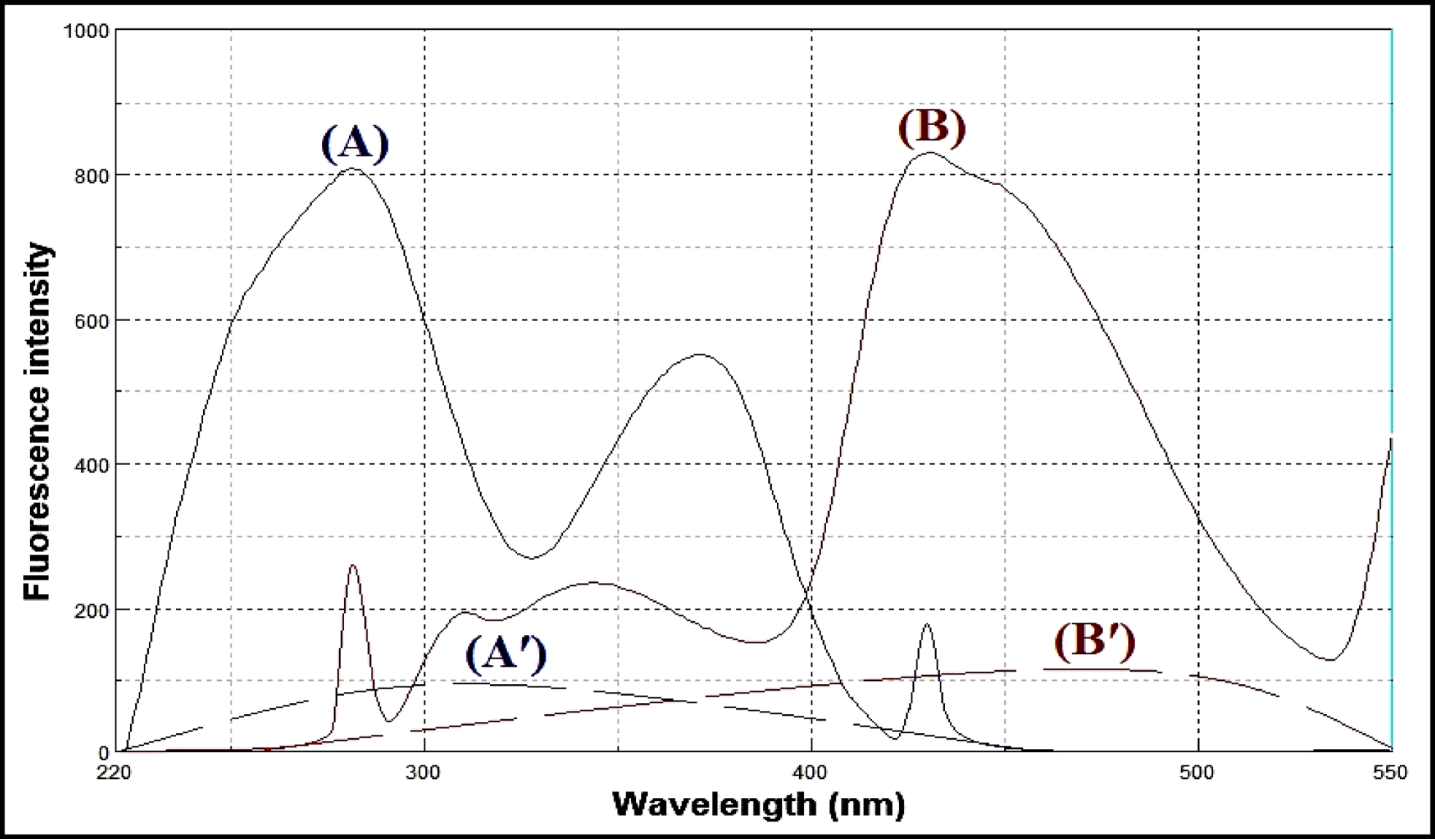
(**A**) Excitation, (**B**) emission and (**A**′, **B**′) blank spectra of lacidipine (250 ng/mL).

**Fig. 4:**
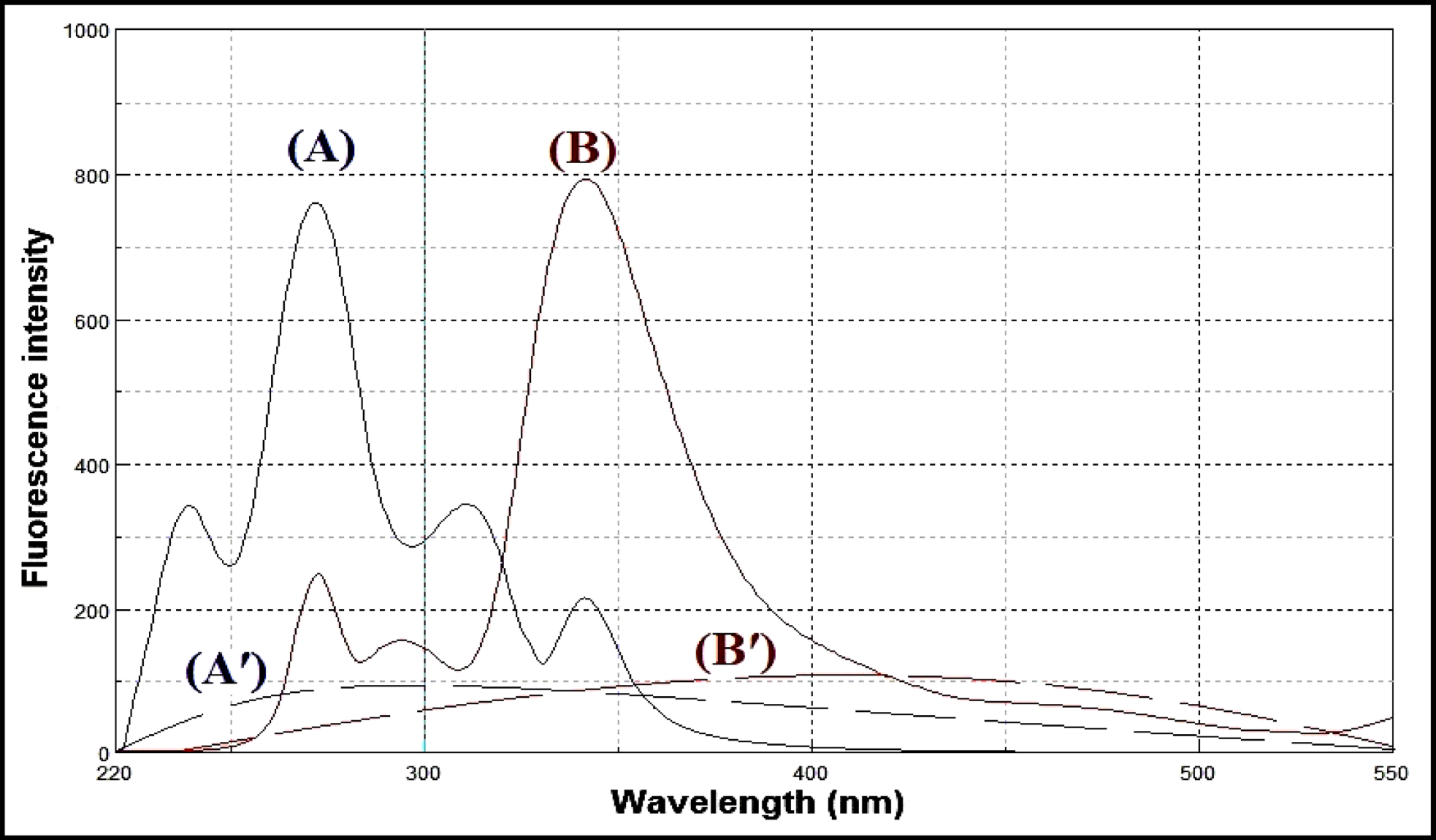
(**A**) Excitation, (**B**) emission and (**A**′, **B**′) blank spectra of lacidipine degradation product (250 ng/mL).

**Fig. 5:**
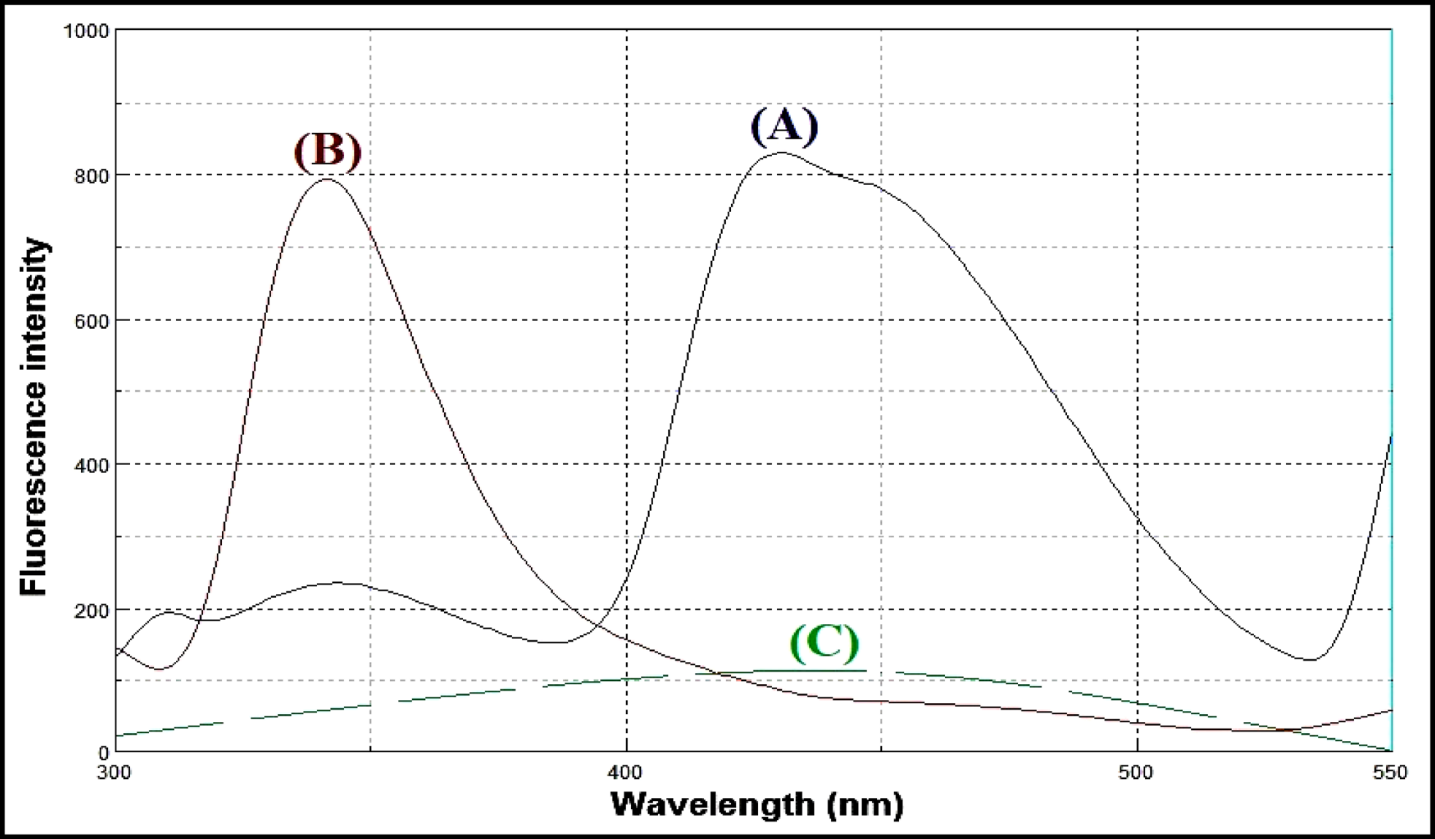
Emission spectra of (**A**) lacidipine (250 ng/mL), (**B**) lacidipine degradation product (250 ng/mL) and (**C**) blank.

**Fig. 6:**
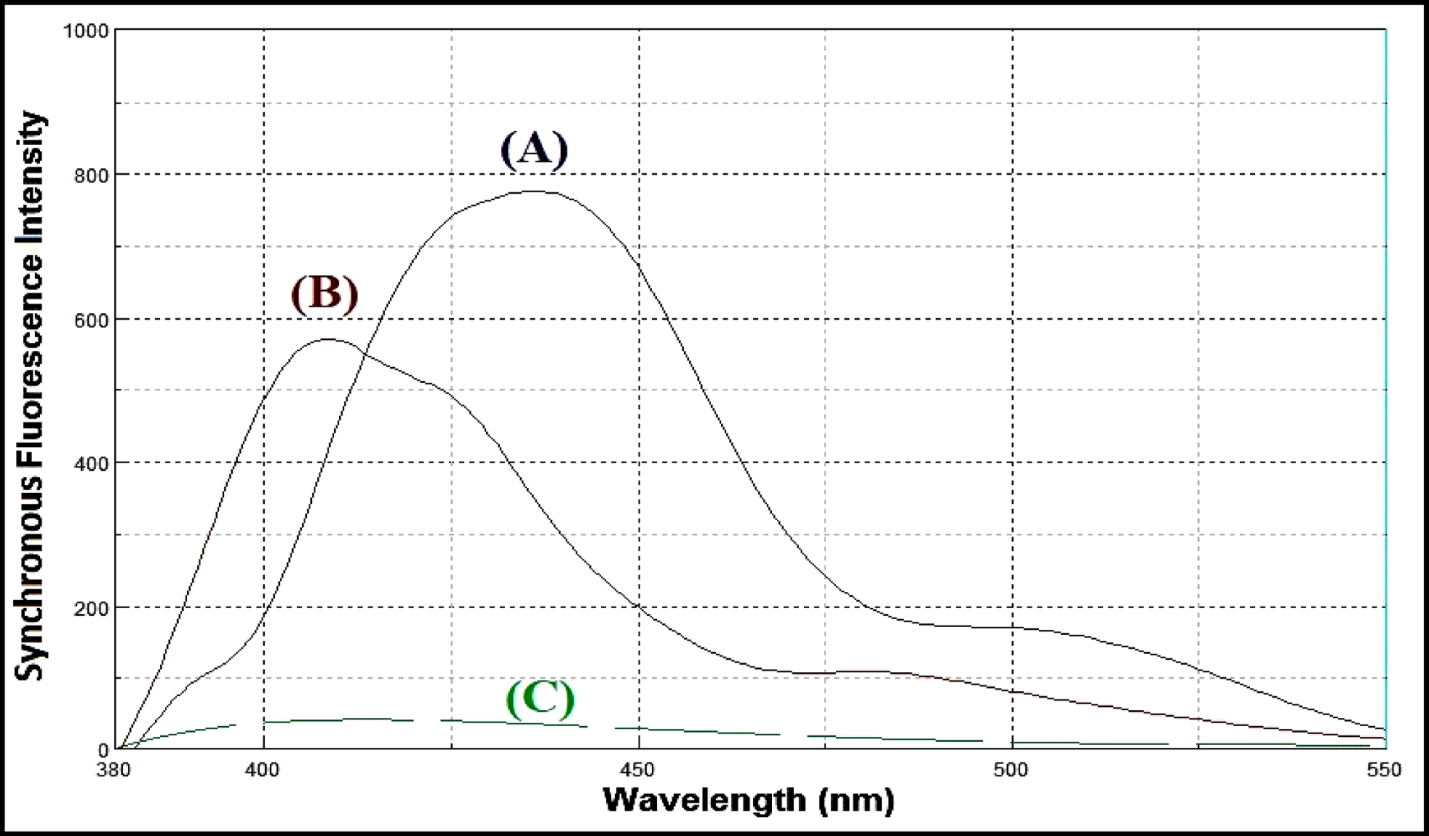
Synchronous fluorescence spectra of (**A**) lacidipine(250 ng/mL), (**B**) lacidipine degradation product (250 ng/mL) and (**C**) blank, using Δλ = 160 nm.

**Fig. 7:**
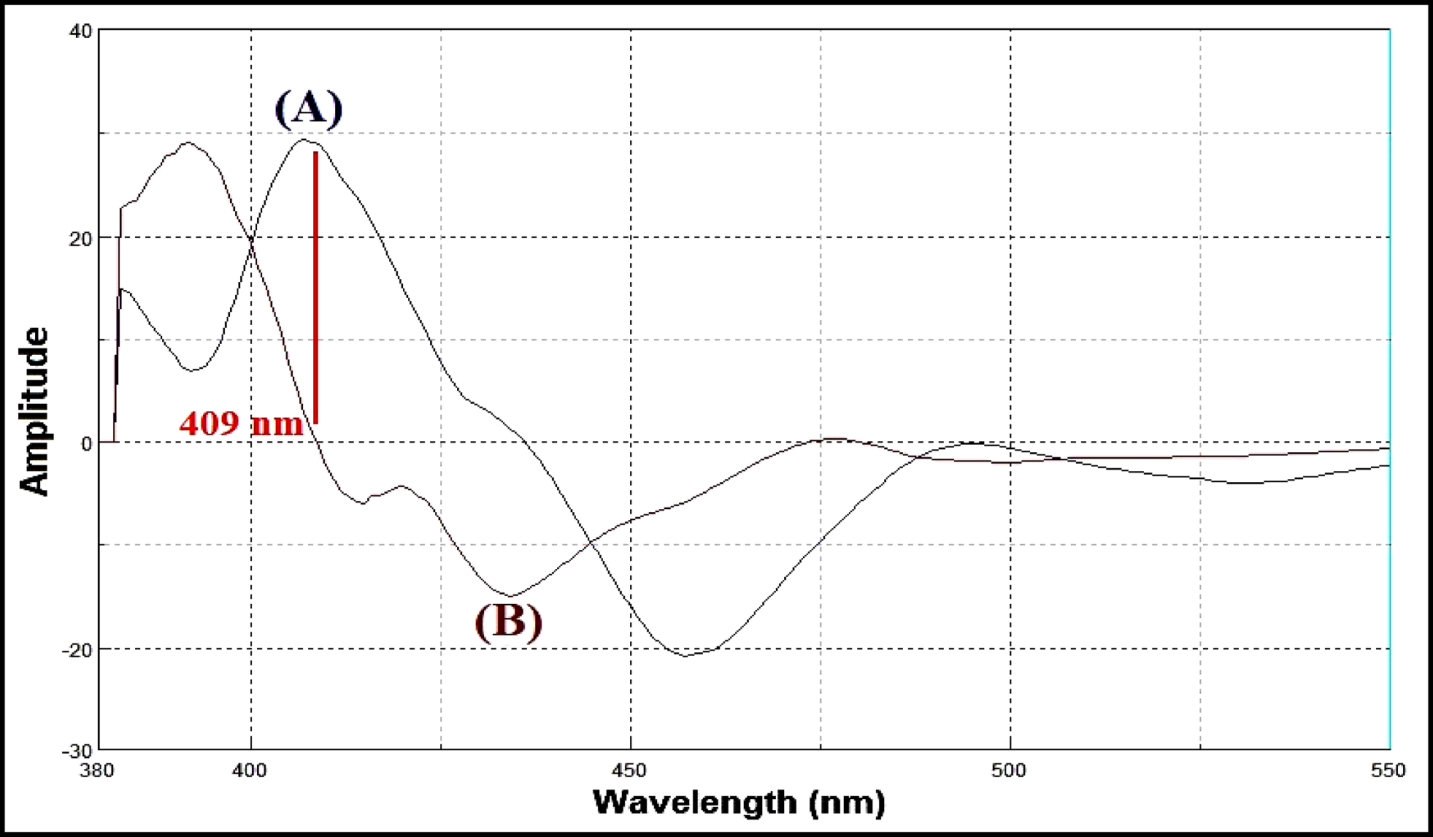
First derivative synchronous fluorescence spectra of (**A**) lacidipine(250 ng/mL) and (**B**) lacidipine degradation product (250 ng/mL) using Δλ = 160 nm.

**Fig. 8:**
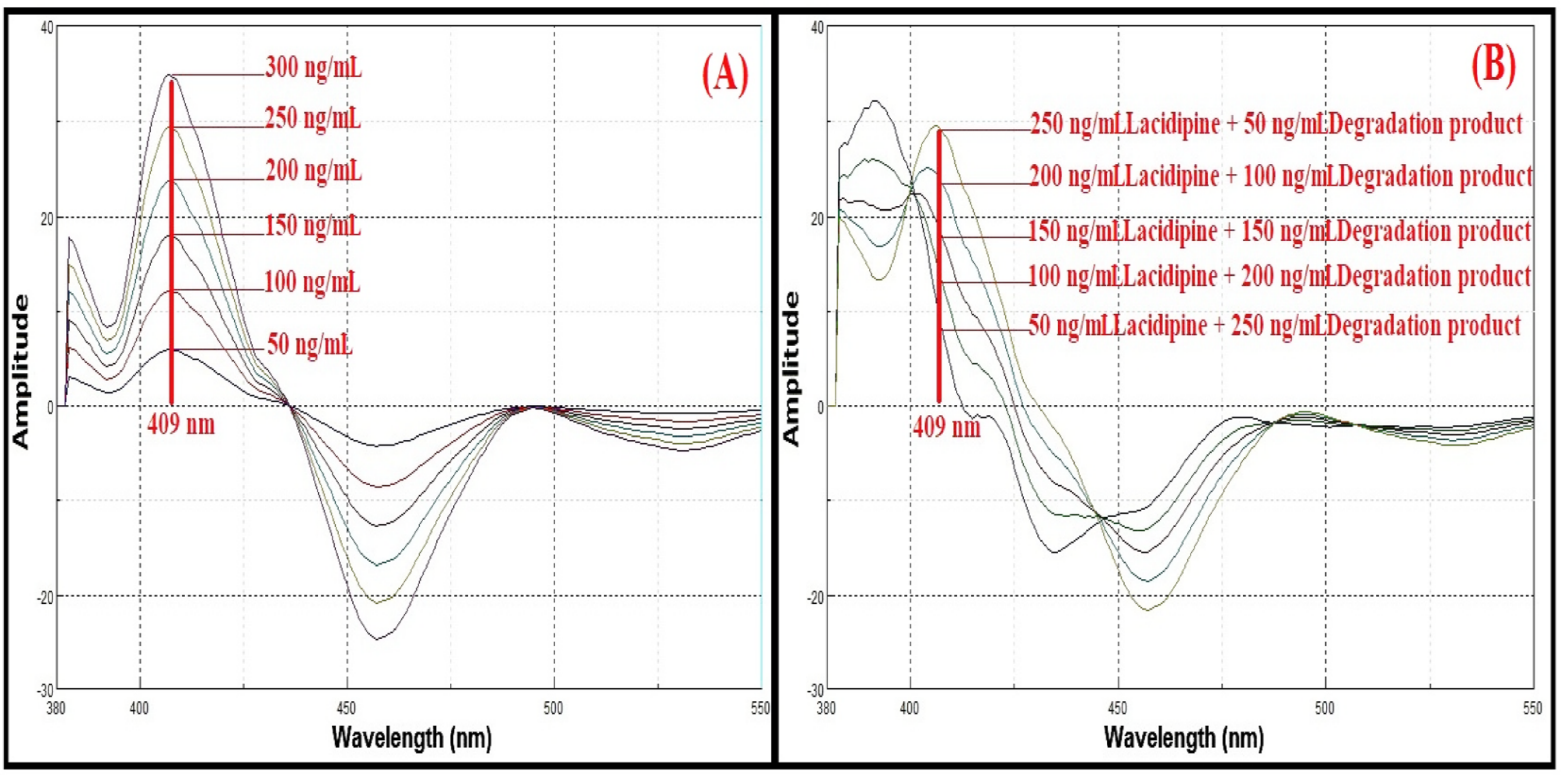
First derivative synchronous fluorescence spectra at 409 nm (**A**) different concentrations of lacidipine (50–300 ng/mL). (**B**) Determination of five component mixtures of lacidipine and degradation product in spiked human plasma.

Lacidipine exhibits native fluorescence with a maximum excitation wavelength at 281 nm and an emission maximum at 430 nm, as shown in Fig. 3. The acid-induced degradation product of lacidipine displays its own fluorescence profile, with excitation at 272 nm and emission at 341 nm, illustrated in Fig. 4. However, the emission spectra of lacidipine and its degradation product show a significant overlap, as demonstrated in Fig. 5, making it difficult to selectively quantify lacidipine in the presence of its degradation product using conventional fluorescence measurement. To resolve this overlap and improve spectral selectivity, synchronous fluorescence scanning was performed using a constant wavelength difference (Δλ = 160 nm). Under these conditions, lacidipine and its degradation product produced distinct synchronous fluorescence signals with enhanced resolution, as presented in Fig. 6. To further improve selectivity and minimize spectral interference, first derivative synchronous fluorescence spectra were applied. This approach allowed for the selective determination of lacidipine at 409 nm, with minimal interference from its degradation product, as clearly observed in Fig. 7.The applicability of the proposed method for determination of lacidipine in spiked plasma and complex mixtures was confirmed by measuring a series of plasma-spiked samples and synthetic mixtures, as shown in Fig. 8.

### Confirmation of degradation product

The degradation of lacidipine was observed by heating under reflux with 1 N hydrochloric acid for 10 h, to confirmation of degradation product TLC, IR^1^,H NMR and mass spectrometry techniques were used.

#### Confirmation of degradation product using TLC

Separation was done by using mobile phase system consists of (toluene: acetone: methanol: ammonia 25%) (60:20:6:2, by volume), although retention factor (R_f_) for lacidipine was 0.79 but for its acid induced degradation was 0.92.

#### Confirmation of degradation product using IR

In IR spectrum of lacidipine a peak for carbonyl group of ester bond was observed at 1631.82 cm^−1^ and peak at 1196.49 cm^−1^ due to (C-O-C) in ester linkage as in Fig. 1S, but in spectrum of degradation product as in Fig. 2S the peak of carbonyl group (C = O) was shifted to 1789.53 cm^−1^ and disappearance of ester linkage (C-O-C) peak which indicate the cleavage of ester linkage and formation of (-COOH) carboxyl group.

#### Confirmation of degradation product using^1^HNMR

Spectra of pure lacidipine as in Fig. 3S and its degradation product in dimethyl sulfoxide (DMSO) showed: δ 8.87 (s, 1 H, NH), 8.14–8.17 (d, J = 14.5 Hz, 1 H, vinylic H), 7.37–7.50 (m, 4 H, Ar-H), 6.37–6.40 (d, J = 14.5 Hz, 1 H, vinylic H), 4.52 (s, 1 H, CH), 4.21–4.25 (m, 4 H, CH_2_), 2.21(s, 6 H, CH_3_), 1.48 (s, 9 H, CH_3_), 1.35–1.38 (m, 6 H, CH_3_). While the degradation product in (DMSO) as in Fig. 4S, showed: δ 11.19 (s, 1 H, OH), 9.94 (s, 2 H, OH), 8.95 (s, 1 H, NH), 8.51–8.54 (d, J = 15.2 Hz, 1 H, vinylic H), 7.41–7.48 (m, 4 H, Ar-H), 6.27–6.30 (d, J = 15.2 Hz, 1 H, vinylic H), 4.67 (s, 1 H, CH), 2.26 (s, 6 H, CH_3_). The appearance of 3 protons of 3 (-COOH) carboxyl groups at 11.19 ppm indicates the cleavage of ester linkage and formation of 3 (-COOH) carboxyl groups and disappear a triplet signal 1.3 ppm which indicate cleavage of ester.

#### Confirmation of degradation product using mass spectrometry

Molecular ion peak for lacidipine was obtained at m/z = 455 as in Fig. 5S but ion peak for its degradation at m/z = 343 as in Fig. 6S.

### Experimental parameters optimization

#### Effect of constant wavelength difference (Δλ selection)

Δλ was tested at 10 nm intervals by experimental trials it was found that Δλ = 160 nm obtain best result for separation of lacidipine from its acid induced degradation.

#### Effect of surfactant type

Undoubtedly impact of surfactant on fluorescence intensity so trials were done to choose the best one by using 1 mL 0.5% w/v solution of each following surfactants; tween-80, anionic surfactant (SDS), (MC), cationic surfactant (CTAB), β-cyclodextrin (β‐CD) and without it, tween-80 was shown to enhance the native fluorescence of lacidipine as in Fig. 7S.

#### Effect of the volume of Tween-80

Different volume of surfactant effect on synchronous fluorescence intensity so after trial experiments on different volumes the optimum volume of tween-80 was chosen to enhance intensity was 1 mL as in Fig. 8S.

#### Effect of diluting solvent

**Fig. 9:**
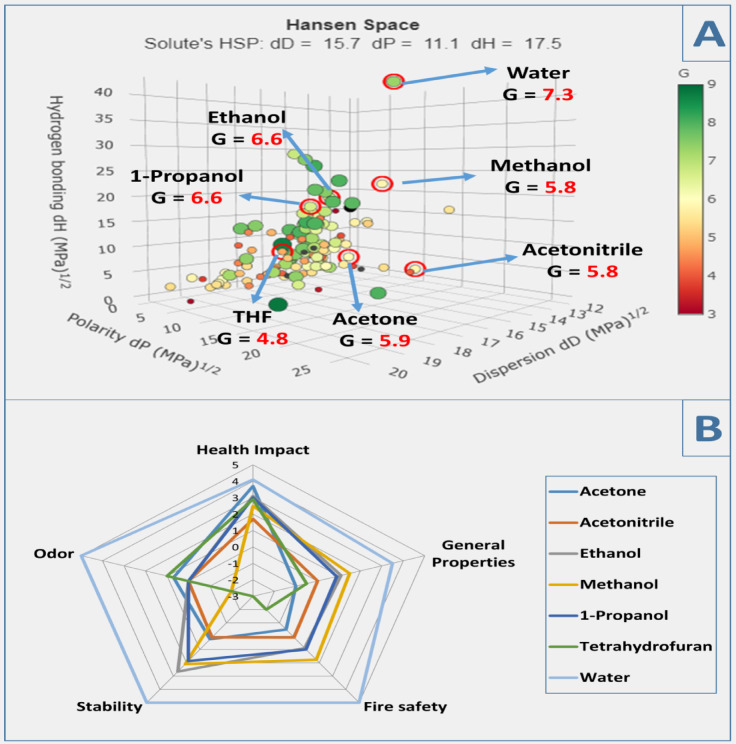
(**A**) Green solvent selection tool. (**B**) Spider diagram based on SDS.

Distilled water, ethanol, methanol, acetone, tetrahydrofuran, 1-propanol and acetonitrile were tested, and by experiment water show high synchronous fluorescence intensity while acetone show low intensity as in Fig. 9S, but intensity of rest of solvent between low and high,

#### Effect of pH

The effect of pH has prominent impact on intensity of synchronous fluorescence, so 1.5 mL of Britton Robinson buffer was added to adjust pH, (pH 5) show high synchronous fluorescence intensity as in Fig. 10S but by increasing pH there was significant reduction on intensity.

#### Effect of buffer volume

The volume of Britton Robinson buffer also effect on synchronous fluorescence intensity, by increasing volume of buffer there was gradually increasing on intensity but when volume of buffer reach to 1.5 mL there was significant peak intensity as in Fig. 11S, but by increasing more there was gradually slight reduction on intensity.

### Validation of the method

According to recommendation of ICH Q2 (R1) about validation of analytical procedures.

#### Linearity and range

The linearity of the proposed method was assessed by analyzing lacidipine standard solutions in the concentration range of 50 to 300 ng/mL. A calibration graph was constructed by plotting the peak amplitude of the first derivative synchronous fluorescence spectra at 409 nm against the corresponding drug concentrations. The regression plot showed excellent linearity, with a correlation coefficient (r²) of 0.9997, indicating a strong linear relationship between concentration and response Fig. 12S. The corresponding regression parameters are provided in Table 1. The linearity was further confirmed by the regression equation (y = 0.1138x + 0.4045) and the correlation coefficient (r² = 0.9997), as shown in Table 1.

**Table 1:** Regression and validation data for the determination of lacidipine by the proposed first derivative synchronous spectrofluorimetric procedure. ^a^ the peak amplitude of the first derivative of synchronous fluorescence spectra.^b^ concentration of Lacidipinein ng/ml.^c^ average of nine determinations (three concentrations repeated three times).^d^ %RSD of nine determinations (three concentrations repeated three times).^e^ average of three determinations.

Parameters	Proposed method
Wavelength (nm)	409
Concentration range (ng/mL)	50 ─ 300
LOD (ng/mL)	14.51
LOQ (ng/mL)	43.97
**Regression Equation** **-** Slope (*b*) **-** Intercept (*a*)	y = 0.1138x + 0.4045 0.1138 0.4045
Coefficient of determination (r^2^)	0.9997
Accuracy (% R)^c^	100.21
**Precision**^d^ **(% RSD)** **-**Repeatability **-**Intermediate precision	1.34 1.12
**Robustness (% R**^e^ ±**% RSD)** **-** Δλ(± 1 nm) **-** pH (± 0.1) **-** Britton Robinson buffer volume (± 0.1 mL)	98.53 ± 0.92 99.47 ± 1.63 101.25 ± 1.21

The range of the method, defined according to ICH Q2 (R1) as the interval between the highest and lowest concentration levels where acceptable accuracy, precision, and linearity were achieved, was established as 50–300 ng/mL. This range was confirmed through experimental validation of both intra-day and inter-day precision and accuracy studies, as summarized in Table 1.

#### Limit of quantification (LOQ) and limit of detection (LOD)

LOQ and LOD had been calculated and in Table 1 results were tabulated.

#### Robustness

Minor changes did not have any clear effect on the intensity of synchronous fluorescence, and the %RSD of the responses was < 2%, confirming the robustness of the procedure as in Table 1.

#### Accuracy

Percent recovery (%R) of 3 different concentration levels were measured (100 ng/mL, 150 ng/mL, 250 ng/mL), each concentration was measured 3different times through 1 day (intraday), so accuracy which equal mean % recovery equal 100.21 as in Table 1.

#### Precision

Calculation of precision not only depend on measuring 3 different concentration levels in one day but also measuring these concentrations at 3 different times through 3 different days (interday), if mean %R and SD calculated on 3 concentrations through 3 different times through 1 day (intraday) precision it called repeatability, but if mean %R and SD calculated on 3 concentrations through 3 different days (interday) precision called Intermediate as in Table 1.

#### Specificity

The specificity of the proposed method was evaluated by testing lacidipine in the presence of its acid degradation product. Two experimental approaches were used:

In the first approach, mixtures containing different concentrations of lacidipine and its degradation product were prepared and analyzed using the proposed first derivative synchronous spectrofluorimetric method. The successful selective determination of lacidipine in these mixtures is shown in Table 2. In the second approach, the standard addition technique was applied to tablet samples to assess the method’s ability to recover lacidipine in the presence of pharmaceutical excipients. The results, presented in Table 3, confirmed the accuracy and selectivity of the method in complex matrices. No interference was observed from the degradation product or from common excipients, indicating that the proposed method is specific for lacidipine.

**Table 2:** Determination of lacidipine in mixtures with its degradation product by the proposed first derivative synchronous spectrofluorimetric procedure.

Intact (ng/ml)	Degradation product (ng/mL)	Degradation product (%)	Intact found (ng/mL)	%Recovery of intact
250	50	16.67	249.67	99.87
200	100	33.33	196.96	98.48
150	150	50	148.78	99.19
100	200	66.67	98.06	98.06
50	250	83.33	50.96	101.92
Mean ± %RSD				99.50 ± 1.53

**Table 3:** Recovery study of lacidipine by standard addition technique using the proposed first derivative synchronous spectrofluorimetric in Lacirex tablet.^a^ average of five determinations. ^b^ average of three determinations.

Pharmaceutical taken (ng/mL)	Pharmaceutical found^a^ (ng/mL)	Pure added (ng/mL)	Pure found^b^ (ng/mL)	Pure recovery (%*R*)
100	98.96	50	49.24	98.48
		100	100.09	100.09
		150	148.69	99.13
		200	197.34	98.67
Mean ± %RSD				99.09 ± 0.72

### Environmental profile of the proposed method

#### Green solvent selection tool (GSST)

Selection of green solvent is one of the main essential steps to maintaining sustainability, some of solvent used in industry and labs have problem issues for environment and health so it is time for using green solvent, recently the pharmaceutical corporations as Pfizer, AstraZeneca, GSK and Sanofi don’t use any solvent except with special specifications which maintain sustainability^31^GSST provide service to select solvent with low environmental and health risks, 3 parameters of solvent are described in 3D Hansen solubility space as in Fig. 9. Theses parameters are polarity, hydrogen bonding and dispersion, color and size of sphere related to characters and impact on environment, the color range from dark green (good solvent) to dark red (bad solvent) and values of G score values range from 10 (good solvent) to 1(bad solvent), for example water has large dark green sphere which indicating a good green solvent with high G score but carbon disulfide has low dark red sphere which indicating bad solvent.

#### Solvent’s spider diagram based on safety data sheet (SDS)

A spider diagram as in Fig. 9 is a visually description of quantitative variables, in proposed case these variables are health impact, stability, fire safety, general properties and odor^32^each variable have subgroups and according the data score value was determined from − 5 to + 5 as shown in Table 1S.

#### Complex GAPI

CGAPI is advanced tool for evaluation the greenness of the analytical procedure, it provide service for researchers to maintain sustainability to their analytical procedures, CGAPI is a modern version than GAPI due to it include process related to steps before analysis and it provide more information through assessment, it depend on visual colored presentation each color has significant mean, green color indicate the method has low impact, yellow color mean medium impact, but red color mean bad impact on environment^33^ as in Table 4.

**Table 4:**
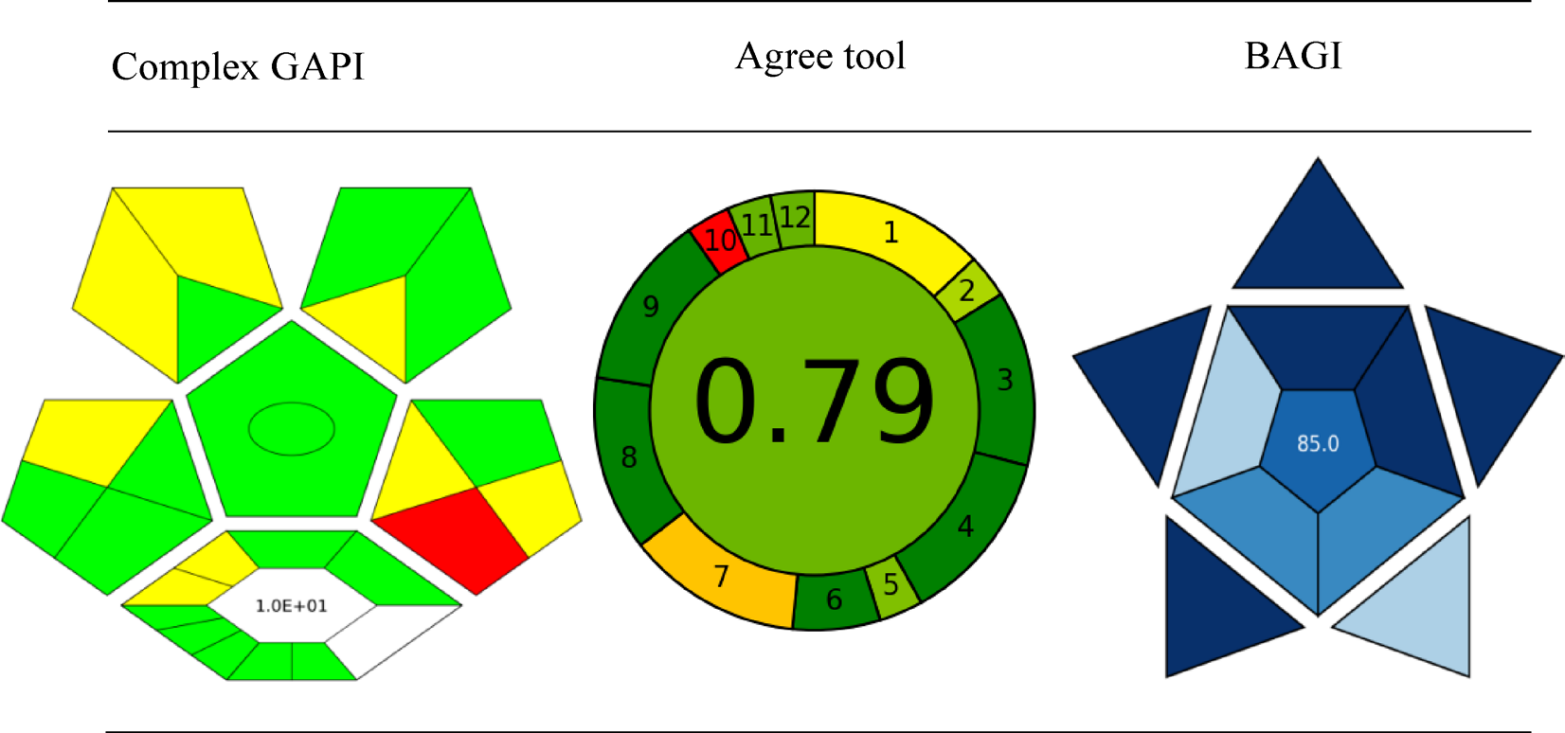
Greenness and blueness evaluation for this method.

#### Agree tool

This tool used to evaluate suitability of procedures of analytical chemistry for maintaining sustainability and evaluation for its greenness, it provide visual pictogram and numerical value that describe the greenness of a method, Agree tool not only associated with procedures but also it concern with reagent, energy consumption and waste product^34^its features based on the twelve principles of green analytical chemistry, each of 12 input variables was transformed in a scale in 0–1 range as in Table 4.

#### BAGI

BAGI convert focus from environmental impact (green chemistry) to applicability and practically (white chemistry), due to importance of analysis in pharmaceutical industry, food, and environmental monitoring BAGI reduce gap between real world application and theoretical analytical procedure^35^this tool has significant effect on encourage development of analytical procedures as in Table 4.

## Application to finished pharmaceutical formulation

The lacidipine in 4 mg Lacirex tablets had been determined by the proposed method, the percent recovery which obtained by the proposed method seem to be satisfactory results when comparing with label claim, and there was no interference from additives and excipients as the result of standard addition techniques as in Table 3.

## Analysis of spiked human plasma

The quantification of lacidipine in spiked human plasma was based on its own calibration curve, which was generated using plasma matrix-matched standards. The resulting regression equation (y = 0.1138x + 0.4045) was used throughout the plasma analysis to ensure accurate results and avoid bias from matrix interference as shown in Table 6. Due to sensitivity of spectrofluorimetric technique monitoring lacidipine at therapeutic levels in spiked plasma samples was done successfully, recovery of lacidipine in spiked human plasma by the first derivative synchronous spectrofluorimetric method were tabulated in Table 5, plasma C_max_ was found 230.68 ± 20.21ng/mL^36^ which exceeding the respective lower limit of detection (LLOD) of drug, according to the FDA guidelines acceptable % recovery of spiked human plasma samples fall within ranges as in Table 6. Five component mixtures of lacidipine and its degradation product were measured by the proposed method at 409 nm in spiked human plasma as in Fig. 9.

**Table 5:** Determination of Lacidipinein spiked human plasma by the proposed first derivative synchronous spectrofluorimetric method.* average of five determinations.

Added (ng/mL)	Found (ng/mL)	*Recovery %
50	46.64	93.29
100	95.26	95.26
150	141.09	94.06
200	192.87	96.43
250	243.84	97.54
**Mean ± %RSD**		95.31 ± 1.81

**Table 6:** Analysis of quality control samples for lacidipine in by the proposed first derivative synchronous spectrofluorimetric method.*average of five determinations.^a^This calibration curve was constructed using spiked human plasma samples following complete sample preparation.

Parameters	Concentration (ng/mL)	*Recovery %
LLOQ	50	95.11
LQC	100	96.91
MQC	150	94.73
	200	95.04
HQC	250	93.85
**Regression equation**	y = 0.1138x + 0.4045^a^	

## Statistical comparison

Statistics had been used to compare reported method with proposed one as in Table 7, by trials under investigation in pharmaceutical dosage form didn’t obtain any statistically significant difference when student’s t-test and F test conducted at 95% confidence level, so the proposed method was precise and accurate.

**Table 7:** Determination of lacidipine in Lacirex tablet by the proposed first derivative synchronous spectrofluorimetric and reported methods * Reported method is a spectrophotometric method which is based on the reaction of lacidipine with ferric chloride, potassium ferricyanide and hydrochloric acid to form a bluish green colored chromogen with an absorption maximum at 740 nm. ** the values in parenthesis are tabulated values of “t” and “F” at (*P* = 0.05).

Parameters	Proposed method	Reported method*
Number of measurements	5	5
Mean % recovery of lacidipine	98.96	100.23
% RSD	0.78	1.15
Variance	0.6	1.33
Student’s *t*-test**	2.056 (2.306)	——
*F*-value**	2.243 (6.388)	——

## Conclusion

Determination of lacidipine and its degradation product was done in spiked human plasma and laboratory prepared mixtures by using spectroflurimetric method in first derivative synchronous mode, the proposed method was fully validated and maintain sustainability, sensitivity was regarded one of the main features of this technique that enable analyzing low concentration of lacidipine and its degradation product in solvents and human plasma.

## Electronic supplementary material

Below is the link to the electronic supplementary material.

Supplementary Material 1

## Data Availability

The data that support the findings of this study are available from the corresponding author, upon reasonable request.
