# Supplementary material for: Green first derivative synchronous spectrofluorimetric determination of lacidipine and its acid degradation product in plasma and mixtures
Source: Sci Rep. 2025 Jul 17;15:25888. doi: 10.1038/s41598-025-11341-y (PMC12267493; doi:10.1038/s41598-025-11341-y)
Supplement: Supplementary file 1 — Supplementary Material 1 [file 41598_2025_11341_MOESM1_ESM.docx]

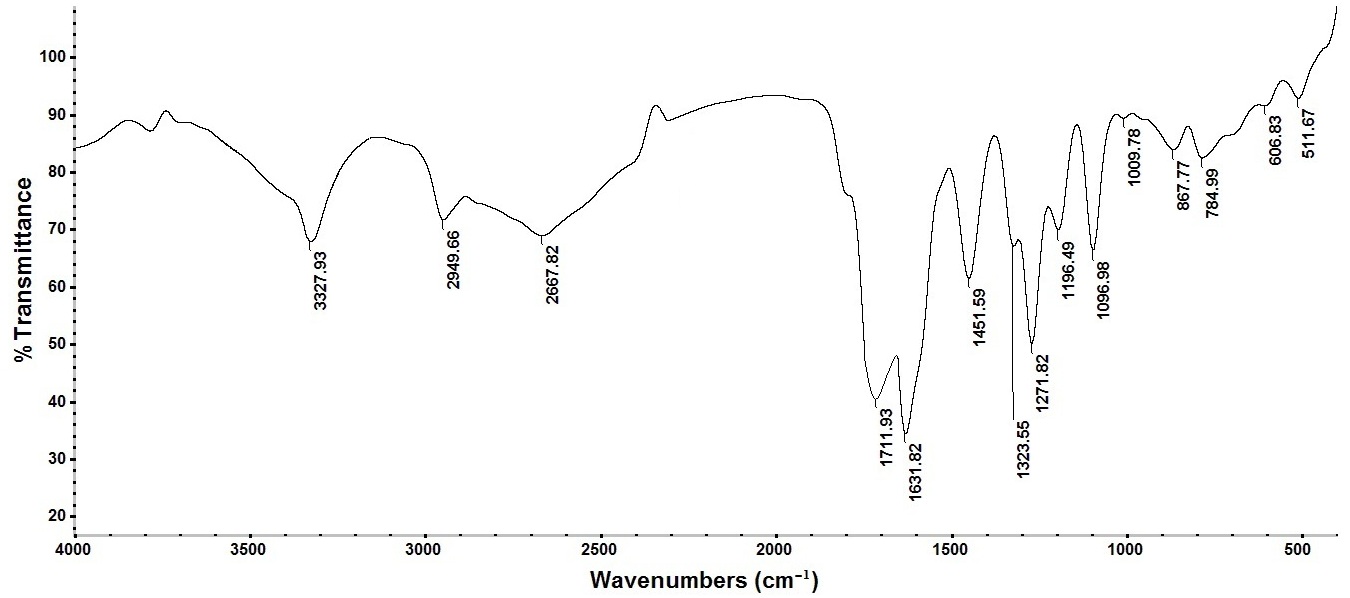


**Fig. 1S: IR spectrum of intact lacidipine.**


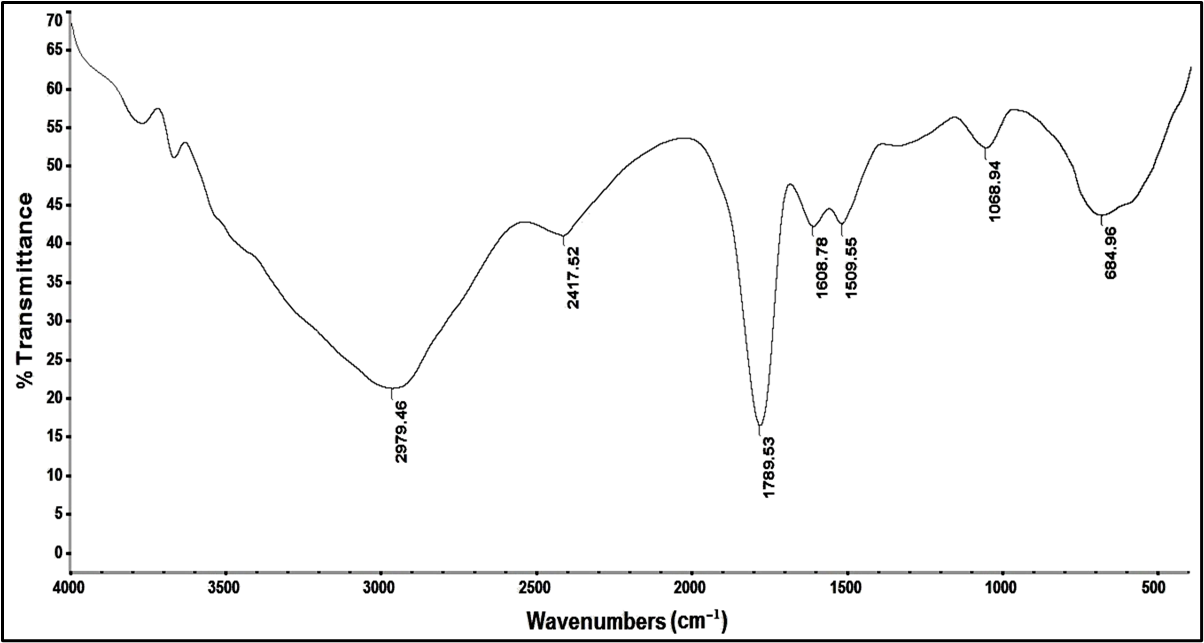


**Fig. 2S: IR spectrum of lacidipine degradation product.**

**
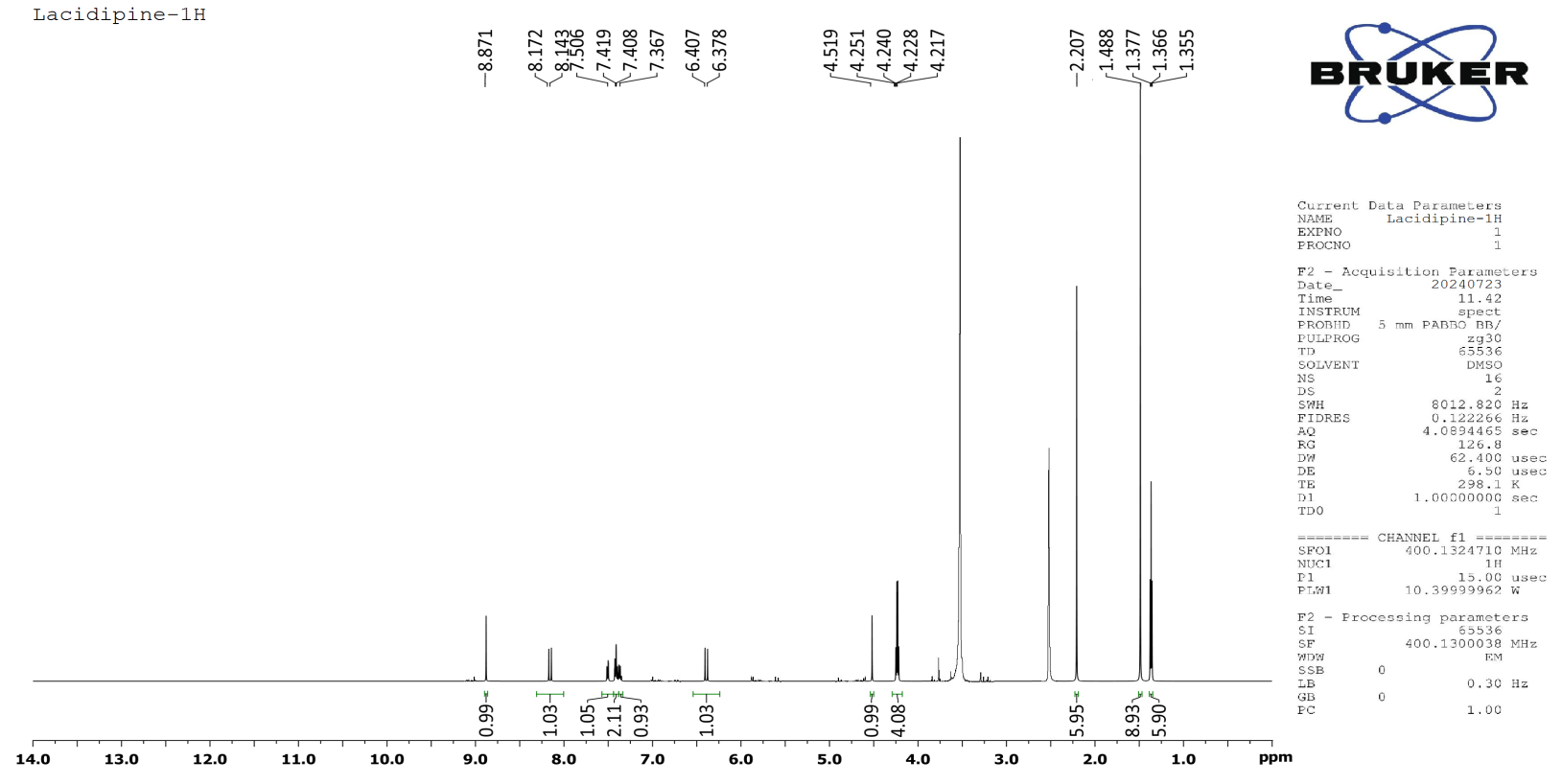
**

**Fig. 3S: ^1^H NMR spectrum of pure lacidipine in (DMSO).**

**
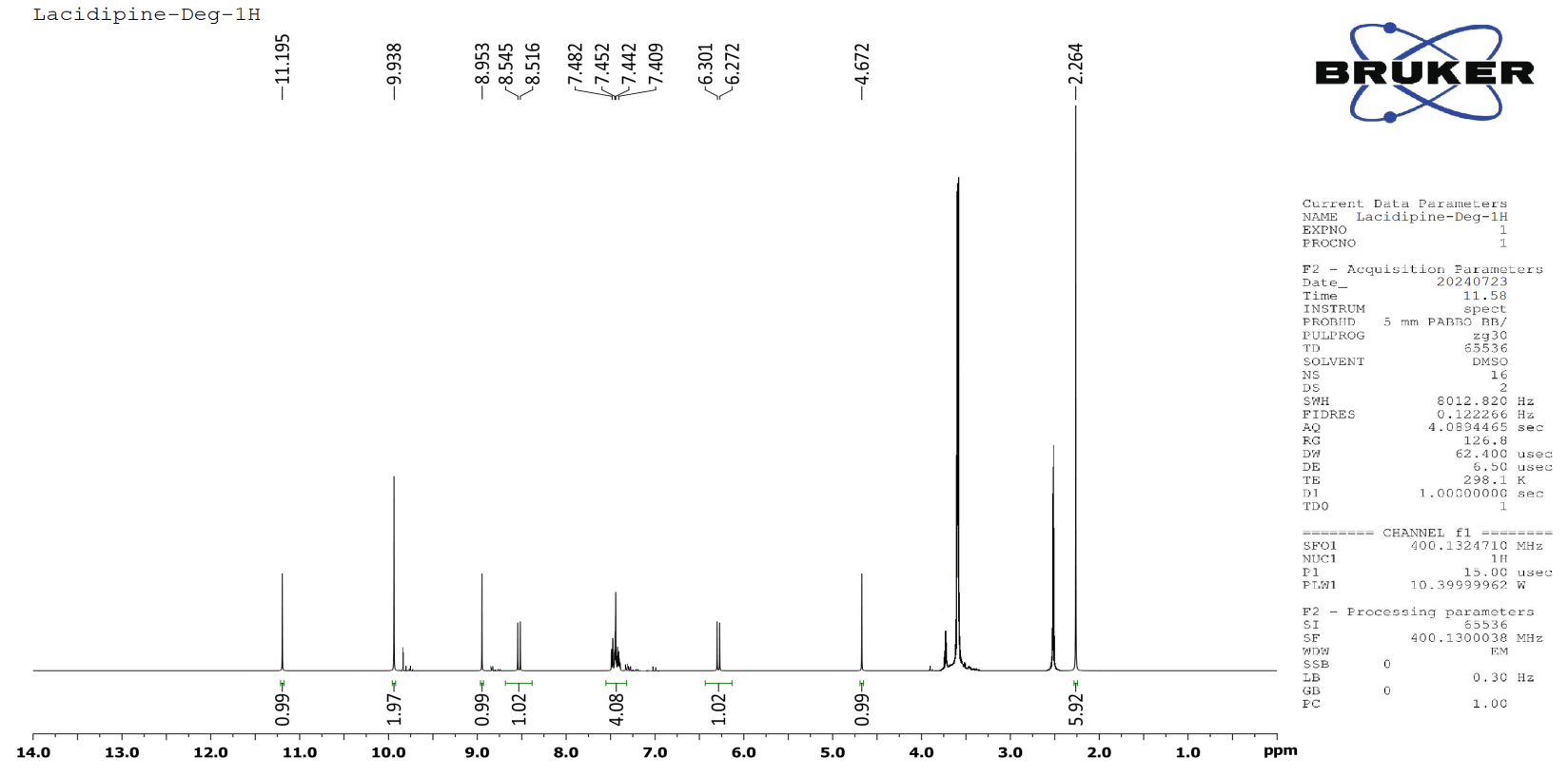
**

**Fig. 4S: ^1^H NMR spectrum of lacidipine degradation product in (DMSO).**


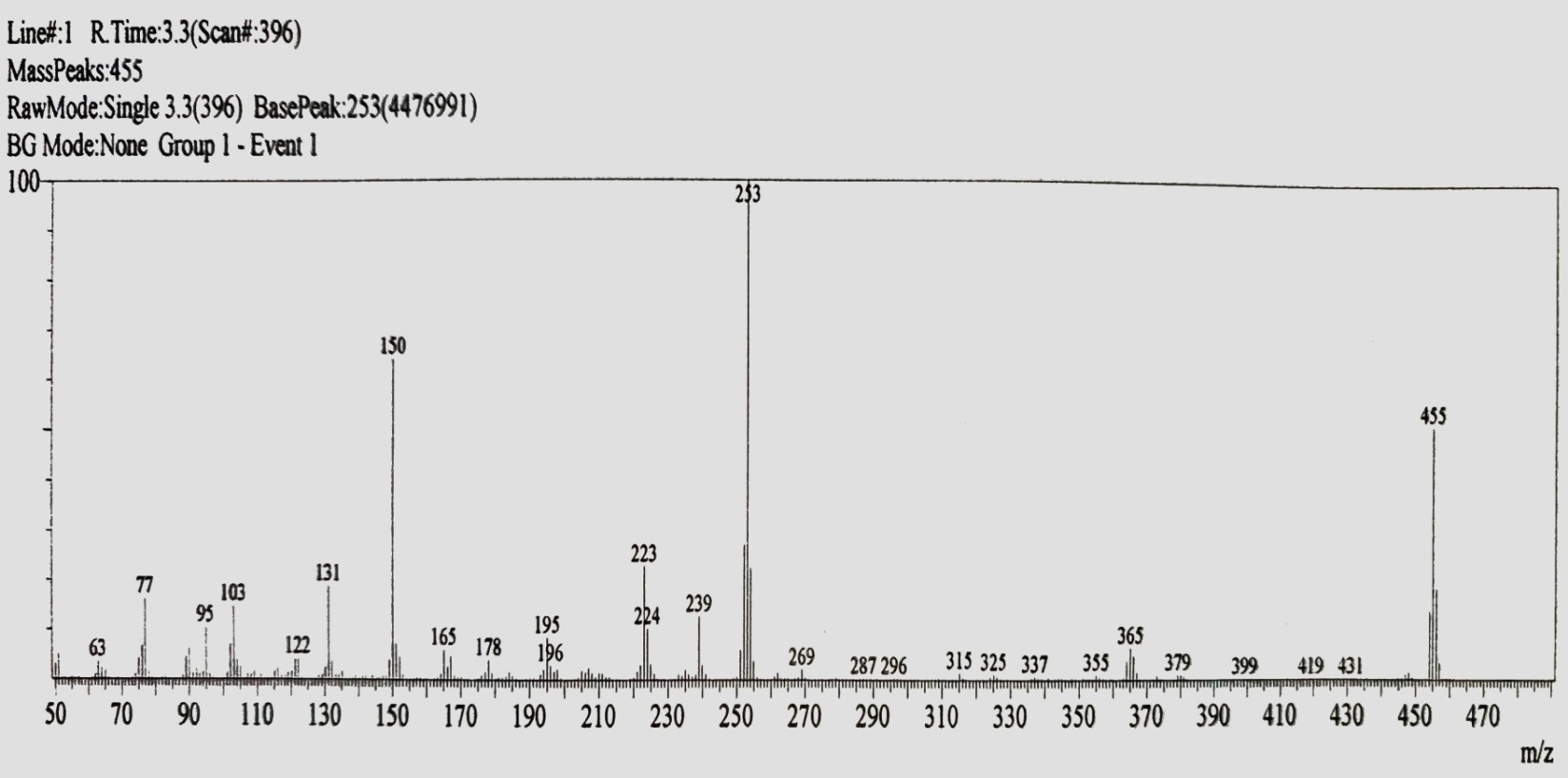


**Fig. 5S: Mass spectrum of intact lacidipine.**

**
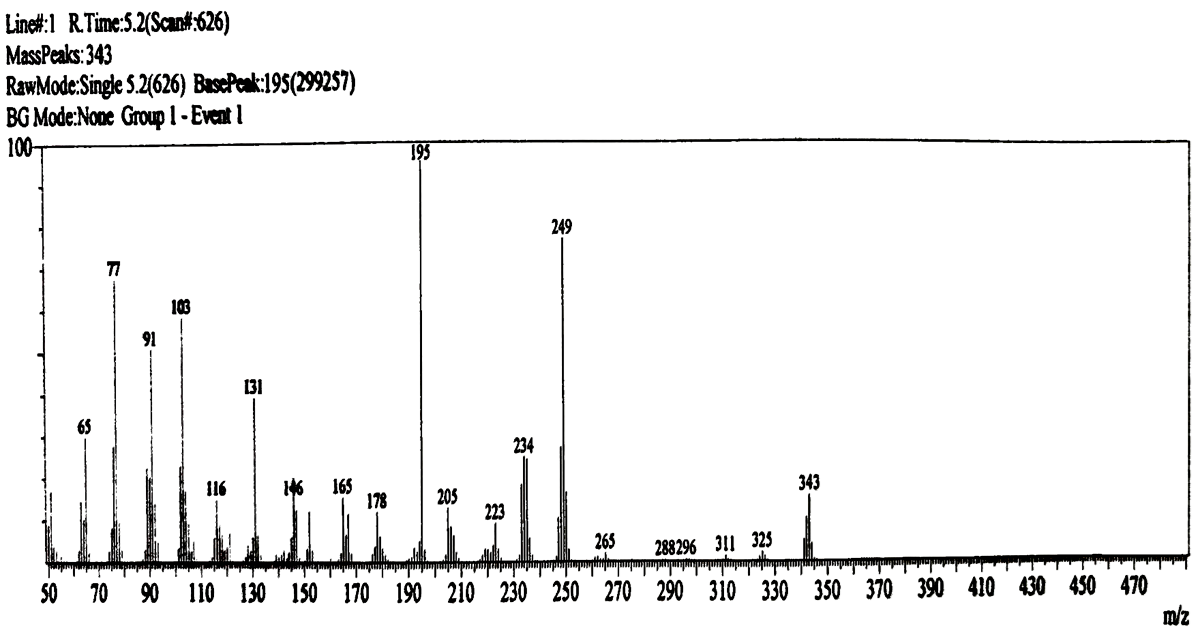
**

**Fig. 6S: Mass spectrum of lacidipine degradation product.**


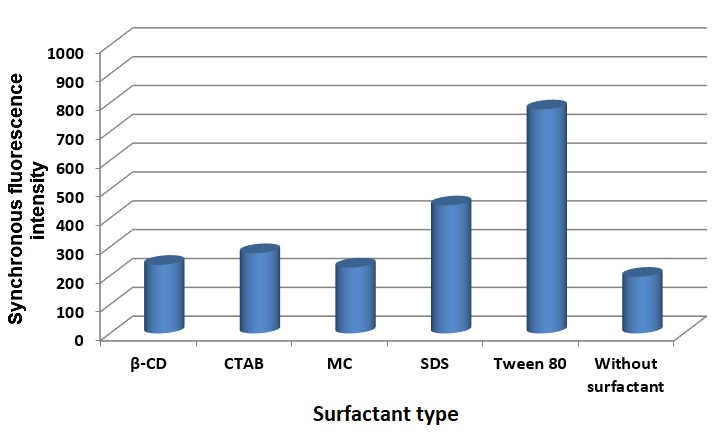


**Fig. 7S: Effect of the surfactant type (1 mL 0.5 % w/v solution of each) on synchronous fluorescence intensity of lacidipine(250 ng/mL).**


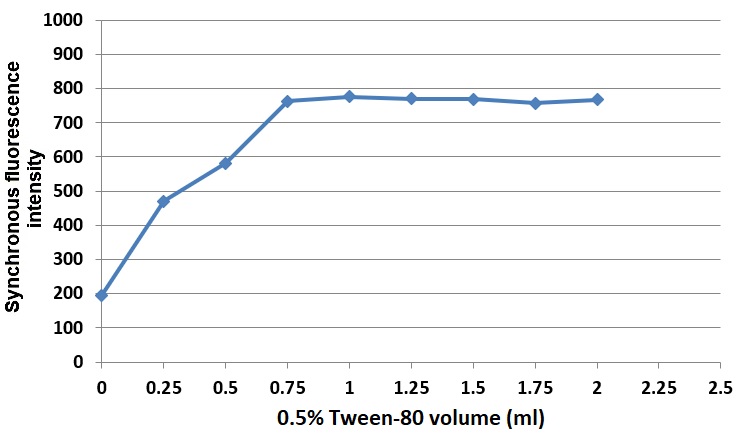


**Fig. 8S: Effect of the volume of 0.5% Tween-80 on synchronous fluorescence intensity of lacidipine (250 ng/mL).**


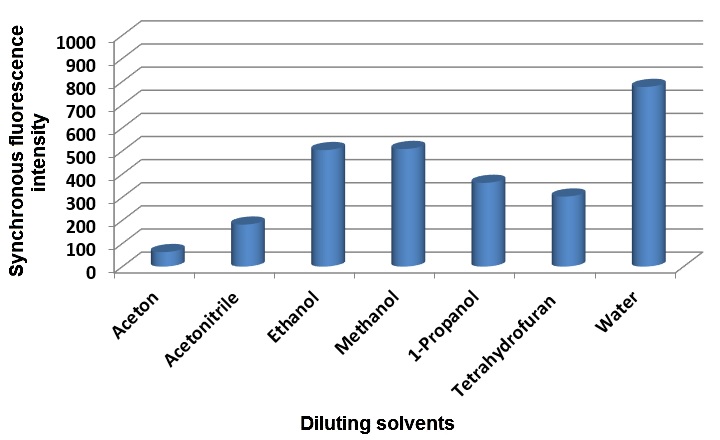


**Fig. 9S: Effect of different diluting solvents on synchronous fluorescence intensity of lacidipine(250 ng/mL).**


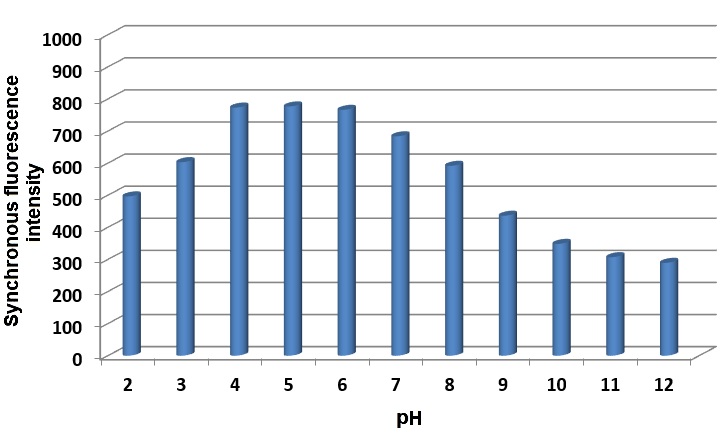


**Fig. 10S: Effect of pH on synchronous fluorescence intensity of lacidipine(250 ng/mL).**


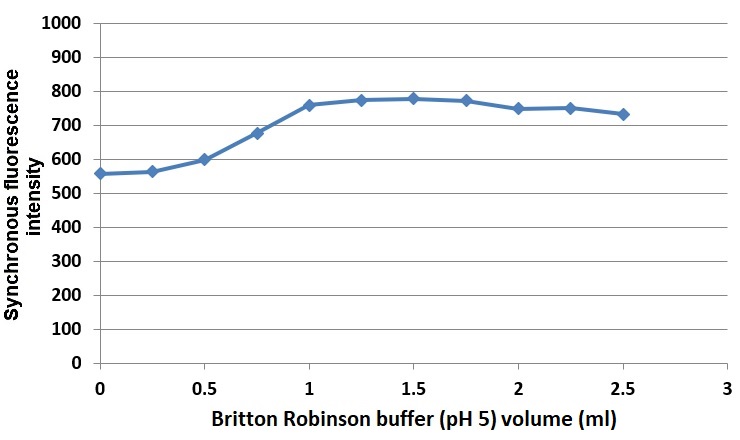


**Fig. 11S: Effect of Britton Robinson buffer volume (pH 5) on synchronous fluorescence intensity of lacidipine (250 ng/mL).**


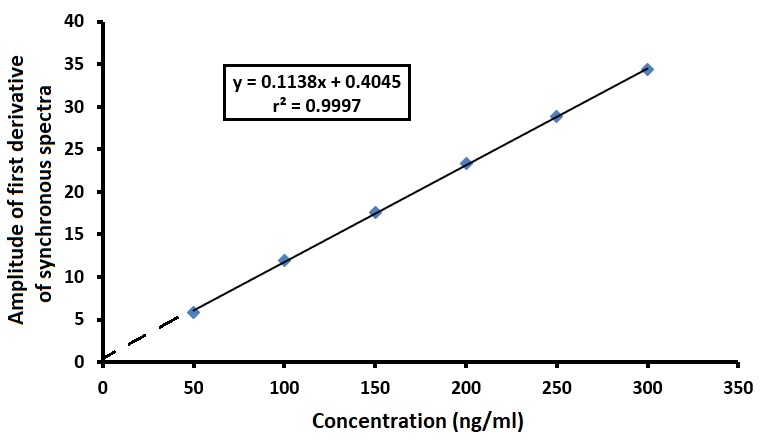


**Fig. 12S: Calibration graph of lacidipine by the proposed first derivative synchronous spectrofluorimetric method.**

**Table 1S: Greenness index table for solvent spider diagram based on SDS**

| **Parameter** | **Acetone** | **Acetonitrile** | **Ethanol** | **Methanol** | **1-Propanol** | **Tetrahydrofuran** | **Water** |
| --- | --- | --- | --- | --- | --- | --- | --- |
| Available information% | 90 | 92 | 77 | 88 | 81 | 95 | 97 |
| Health Impact | 3.7 | 1.7 | 3.1 | 2.5 | 3.005 | 2.9 | 4.1 |
| General Properties | -1 | 0.01 | 1.1 | 1.5 | 0.9 | -0.5 | 3.5 |
| Fire safety | -0.5 | 0.1 | 0.9 | 1.77 | 1 | -2 | 5 |
| Stability | 0.23 | 0.09 | 2.66 | 2.1 | 1.872 | -3 | 5 |
| Odor | 0.7 | 0 | 0 | -2 | 0 | 1 | 5 |
